# Several distance and degree-based molecular structural attributes of cove-edged graphene nanoribbons

**DOI:** 10.1016/j.heliyon.2024.e34944

**Published:** 2024-07-23

**Authors:** S. Prabhu, G. Murugan, Muhammad Imran, Micheal Arockiaraj, Mohammad Mahtab Alam, Muhammad Usman Ghani

**Affiliations:** a Department of Mathematics, Rajalakshmi Engineering College, Thandalam, Chennai 602105, India; b Department of Mathematics, Chennai Institute of Technology, Chennai 600069, India; c Department of Mathematical Sciences, United Arab Emirates University, Al Ain, P. O. Box 15551, United Arab Emirates; d Department of Mathematics, Loyola College, Chennai 600034, India; e Central Labs, King Khalid University, AlQura'a, Abha, P.O. Box 960, Saudi Arabia; f Department of Basic Medical Sciences, College of Applied Medical Science, King Khalid University, Abha 61421, Saudi Arabia; g Institute of Mathematics, Khawaja Fareed University of Engineering & Information Technology, Abu Dhabi Road, 64200, Rahim Yar Khan, Pakistan

**Keywords:** Molecular graph, Topological indices, Nanographene, Nanoribbon

## Abstract

A carbon-based material with a broad scope of favourable developments is called graphene. Recently, a graphene nanoribbon with cove-edged was integrated by utilizing a bottom-up liquid-phase procedure, and it can be geometrically viewed as a hybrid of the armchair and the zigzag edges. It is indeed a type of nanoribbon containing asymmetric edges made up of sequential hexagons with impressive mechanical and electrical characteristics. Topological indices are numerical values associated with the structure of a chemical graph and are used to predict various physical, chemical, and biological properties of molecules. They are derived from the graph representation of molecules, where atoms are represented as vertices and bonds as edges. In this article, we derived the exact topological expressions of cove-edged graphene nanoribbons based on the graph-theoretical structural measures that help reduce the number of repetitive laboratory tasks necessary for studying the physicochemical characteristics of graphene nanoribbons with curved edges.

## 1 Introduction

Graphene has attracted tremendous consideration because of its unprecedented blend of electronic, mechanical, thermal and optical characteristics [1]. Lately, graphene research has been extraordinarily dynamic, and the evolution in the manufacture and portrayal of graphene has been exceptionally critical [2]. Among the numerous qualities of the 2D-material graphene, its high charge-carrier portability is one of the most significant as it permits graphene a guarantee of fantastic execution in field-effect transistors (FETs) [3], [4], [5]. It has quickly grown to prominence in the field of science and technology. Because of its recognized electrical, compound and optical exhibitions, graphene and its subordinates have additionally created enormous interest in analytical chemistry. The vacancy of an energy bandgap, which is a sign of semiconductor materials, prevents these materials from being used in optoelectronic devices [6], [7]. Reducing one of the graphene sheet dimensions till it influences atomic sizes is one way to design a gap opening. Graphene nanoribbons (GNRs) are to bring in the next level of semiconductor applications [8].

**Figure 1:**
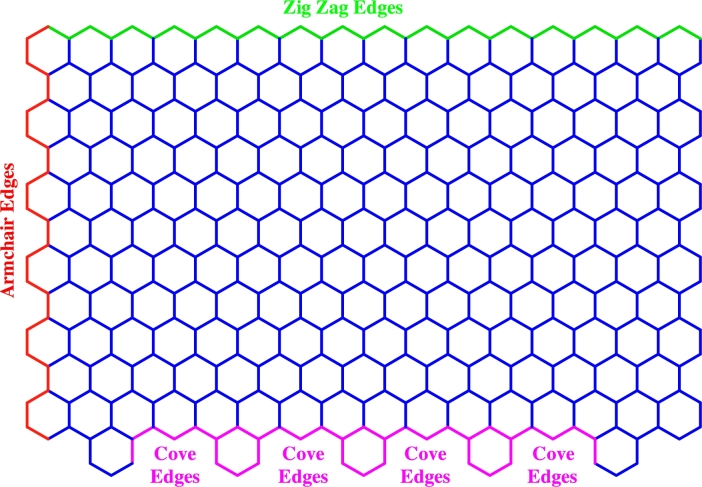
Armchair, zigzag and cove peripheries.

Graphene nanoribbons appear as a cutting edge transporter for improving nano dimensional symptomatic gadgets and medication delivery frameworks because of the exciting and forefront electronic, warm, mechanical and optical properties related with graphene [9], [10]. They are exceptionally evolved graphenes with a broad significance because of their peculiar properties, such as enormous surface territory, upgraded mechanical quality, and improved electro-conductivity. These nanoribbons are the best transporter for anticancer medications and other exceptionally aromatic medications [11]. The semi one-dimensional extended monolayer segments of graphenes have a high length to width proportion. The length and breadth estimations can be used to communicate the dimensions of the GNRs [12]. They are synthetical $sp^{2}$ hybridised carbon structures with a honeycomb grid geometry. The edge structure is the important component in GNR characteristics, with armchair-edge GNRs (AGNRs) semiconducting along width-subordinate bandgaps and zigzag-edge GNRs (ZGNRs) probably appealing owing to their edge-restricted states, which can be turned polarised. Furthermore, armchair and zigzag edge configurations, alternative geometries such as cove edges [13], [14] that result in non-planarity owing to the aversion amid nearby hydrogen molecules [15] can also be considered. See Fig. 1.

**Figure 2:**
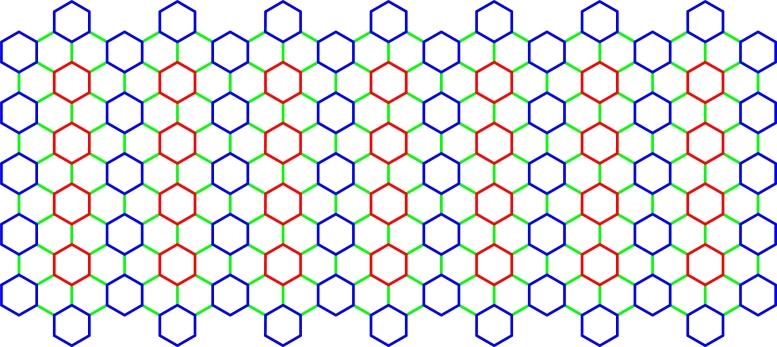
*CGNR*(5,7).

Recently [16], [17], [18], [19], bottom-up synthesis of graphene permitted a cove edged graphene nanoribbons (CGNRs) as shown in Fig. 2 which is the combination of an armchair and zigzag peripheries. This construction was described as structurally explicit and remarkably long (>200nm). Exchanging electron rich and electron-poor subunits tune the frontier orbitals of CGNRs, which are molecularly defined and soluble. In solution-made cove-edged nanoribbons have the added benefit of a strangely shaped, distorted *π* surface which is deeply dissolvable [20]. When properly functionalized, solvent nanoribbons are admirable electron-transporting materials. These structures have been used in various fascinating classes containing electronic [21], chemical [22] and mechanical applications [23].

Cheminformatics assumes a vital part to keep up and accesses the gigantic measure of chemical data created by chemists utilizing a legitimate database. Furthermore, research requires a creative approach for extracting information from data to demonstrate complicated interconnections amid the structure of biological activity and a chemical molecule, as well as the impact of reaction conditions on chemical reactivity [24]. Structure portrayal manages reaction characterization, structure descriptors and searching, molecular modelling, and computer-assisted structure elucidation. The interactions between countless chemical and, in particular, biological facts of substances and their structure are far too complicated to be reliably anticipated using fundamental principles. Structure descriptors (Topological indices) must be determined for the structures of a dataset [25]. At that stage, data analysis or a model-building method must be used to create a scientific model for the connection between the topological indices and the explored property. For the two stages, a lot of strategies have been created. A large group of techniques, including in the large numbers, for computing structure descriptors, is accessible [26]. Increasingly more consideration presently moves to the utilization of molecular indices which can be deciphered and consequently give a model that builds experiences into the connection between a compound's structure and properties. The atomic descriptors are helpful in portraying the 1D, 2D, 3D structure, or the sub-atomic surface properties [27]. Furthermore, the representation of chemical molecules is given more attention than just a subatomic characterisation [28].

Inspired by the work of Wiener and Randić, the distance [29], [30], [31], [32] based and degree [33], [34], [35], [36], [37] based indices are investigated by many researchers in the domain of molecular science. Also, several other topological indices for molecular graphs have been detailed. Wiener and Szeged type indices are instances of distance-based topological indices. Schultz and Gutman indices are degree-distance indices, were given this way. For recent research on this topic the reader can refer [38], [39], [40], [41], [42], [43], [44], [45], [46], [47]. There are also connectivity-based indices, such as the generalised connectivity index, Zagreb index, Estrada's ABC index, harmonic, sum-connectivity, and geometric-arithmetic indices, that follow Randić connectivity index. The dermal penetrations and octanol segment coefficients of aromatic compounds have been shown in [48] to correlate strongly with topological connectivity indices. Melting temperatures, boiling points, vapour pressures, chromatographic retention indices, toxicities, reported bioactivities, osmotic coefficients, and diffusion constants, on the other hand, have all been linked to both vertex-degree and edge-based topological indices. For more on the application part of topological indices, the reader can refer [49], [50], [51]. This article presents the mathematical expressions of several distances and degree-based indices of a graphene nanoribbon with cove-type edges.

## 2 Graph-theoretical terminologies

The degree of a vertex $\eta_{G}(a)$ is the number of edges that are connected to it, and the neighbourhood of a vertex *a* is the set of vertices that are adjacent to it, $N_{G}(a)$. Denote $\eta_{G}(a,b)$ as the minimum number of edges that connects a,b∈V(G), which is the formally called distance among them. In the same context, $\eta_{G}(a,f)=\min\{\eta_{G}(a,s),\eta_{G}(a,t)\}$ for a∈V(G) f=st∈E(G) and $D_{G}(e,f)=\min\{\eta_{G}(a,f),\eta_{G}(b,f)\}$ for e,f∈E(G), where e=ab and f=st. If $\eta_{G}(a,b)=d_{G^{\prime }}(a,b)$ for a subgraph $G^{\prime }$ of G, then $G^{\prime }$ is an isometric, and if all the graph geodesic between $a,b\in V(G^{\prime })$ fall completely within $G^{\prime }$, then $G^{\prime }$ is a convex.

$N_{a}(e|G)$ and $M_{a}(e|G)$ are the subset of V(G) and E(G) that are closer to *a*, when compared to *b*, and the cardinal number of these two sets are respectively represented by $n_{a}(e|G)$ and $m_{a}(e|G)$, for an edge e=ab∈E(G). The variables $n_{b}(e|G)$ and $m_{b}(e|G)$ are designated in the same way.

The strength-weighted graph or quotient graph was initially published in [31] and it has since been extensively addressed [52], [53], [54], [55], [56], [57], [58], [59], [60], [61] as $\mathrm{SWG}=(G,(w_{b},s_{b}),s_{e})$ where $w_{b}:V(G_{sw})\to W$, and $s_{b}:V(G_{sw})\to W$ and the $s_{e}:E(\mathrm{SWG})\to W$. In this context, it is alwas true that $d_{\mathrm{SWG}}(a,b)=\eta_{G}(a,b)$, $d_{\mathrm{SWG}}(a,f)=\eta_{G}(a,f)$, $D_{\mathrm{SWG}}(e,f)=D_{G}(e,f)$, $N_{a}(e|\mathrm{SWG})=N_{a}(e|G)$ and $M_{a}(e|\mathrm{SWG})=M_{a}(e|G)$. The cardinality of clossness is calculated by $n_{a}(e|\mathrm{SWG})=\sum_{x\in N_{a}(e|\mathrm{SWG})}w_{b}(x)$, and $m_{a}(e|\mathrm{SWG})=\sum_{x\in N_{a}(e|\mathrm{SWG})}s_{b}(x)+\sum_{f\in M_{a}(e|\mathrm{SWG})}s_{e}(f)$. The computations of $n_{b}(e|\mathrm{SWG})$ and $m_{b}(e|\mathrm{SWG})$ are interpreted in the same way. The degree of a vertex *a* in SWG is estimated by $d_{\mathrm{SWG}}(a)=\sum_{x\in N_{\mathrm{SWG}}(a)}s_{e}(ax)$. When $w_{b}=1$, $s_{b}=0$ and $s_{e}=1$, we construct the significant distance-based TIs of SWG and observe that TI(SWG)=TI(G).

1. **Wiener Type Indices:**
  (1) $$W(\mathrm{SWG})=\sum_{\left\{a,b\}\subseteq V(\mathrm{SWG}\right)}w_{b}(a)w_{b}(b)d_{\mathrm{SWG}}(a,b)$$
  (2) $$W_{e}(\mathrm{SWG})=\sum_{\left\{a,b\}\subseteq V(\mathrm{SWG}\right)}s_{b}(a)\;s_{b}(b)\;d_{\mathrm{SWG}}(a,b)+\sum_{\left\{e,f\}\subseteq E(\mathrm{SWG}\right)}s_{e}(e)\;s_{e}(f)\;D_{\mathrm{SWG}}(e,f)\;\;\;\;\;+\;\sum_{a\in V(\mathrm{SWG})}\sum_{f\in E(\mathrm{SWG})}s_{b}(a)\;s_{e}(f)\;d_{\mathrm{SWG}}(a,f)$$
  (3) $$W_{ve}(\mathrm{SWG})=\frac{1}{2}[\sum_{\left\{a,b\}\subseteq V(\mathrm{SWG}\right)}\{w_{b}(a)\;s_{b}(b)\;+\;w_{b}(b)\;s_{b}(a)\}\;d_{\mathrm{SWG}}(a,b)\;\;\;\;\;+\sum_{a\in V(\mathrm{SWG})}\sum_{f\in E(\mathrm{SWG})}w_{b}(a)\;s_{e}(f)\;d_{\mathrm{SWG}}(a,f)]$$
2. **Szeged Type Indices:**
  (4) $$Sz_{v}(\mathrm{SWG})=\sum_{e=ab\in E(\mathrm{SWG})}s_{e}(e)n_{a}(e|\mathrm{SWG})n_{b}(e|\mathrm{SWG})$$
  (5) $$Sz_{e}(\mathrm{SWG})=\sum_{e=ab\in E(\mathrm{SWG})}s_{e}(e)m_{a}(e|\mathrm{SWG})m_{b}(e|\mathrm{SWG})$$
  (6) $$Sz_{ev}(\mathrm{SWG})=\frac{1}{2}\sum_{e=ab\in E(\mathrm{SWG})}s_{e}(e)[n_{a}(e|\mathrm{SWG})m_{b}(e|\mathrm{SWG})+n_{b}(e|\mathrm{SWG})m_{a}(e|\mathrm{SWG})]$$
  (7) $$PI(\mathrm{SWG})=\sum_{e=ab\in E(\mathrm{SWG})}s_{e}(e)[m_{a}(e|\mathrm{SWG})+m_{b}(e|\mathrm{SWG})]$$
3. **Degree and Distance-Based Type Indices:**
  (8) $$S(\mathrm{SWG})=\sum_{\left\{a,b\}\subseteq V(\mathrm{SWG}\right)}[w_{b}(b)(d_{\mathrm{SWG}}(a)+2s_{b}(a))+w_{b}(a)(d_{\mathrm{SWG}}(b)+2s_{b}(b))]d_{\mathrm{SWG}}(a,b)$$
  (9) $$Gut(\mathrm{SWG})=\sum_{\left\{a,b\}\subseteq V(\mathrm{SWG}\right)}(d_{\mathrm{SWG}}(a)+2s_{b}(a))(d_{\mathrm{SWG}}(b)+2s_{b}(b))d_{\mathrm{SWG}}(a,b)$$

When dealing with distance-based TIs, the cut technique is quite useful [62], [63]. This technique is based on the Djoković-Winkler *Θ* condition, $\eta_{G}(a,c)+\eta_{G}(b,d)\neq \eta_{G}(a,d)+\eta_{G}(b,c)$ for ab,cd∈E(G). The relation *Θ* is symmetric and reflexive, although it is not transitive in general. For convenience, we denote {1,2…l} as $N_{l}$.

We close this section by stating the degree-based topological indices [33], [34], [35], [36], [37] as follows:

• Randić: $R(G)=\sum_{ab\in E(G)}\frac{1}{\sqrt{\eta_{G}(a)\eta_{G}(b)}}$
• Reciprocal Randić: $RR(G)=\sum_{ab\in E(G)}\sqrt{\eta_{G}(a)\eta_{G}(b)}$
• Reduced reciprocal Randić: $RRR(G)=\sum_{ab\in E(G)}\sqrt{\left(\eta_{G}(a)-1)(\eta_{G}(b)-1\right)}$
• First Zagreb: $M_{1}(G)=\sum_{a\in V(G)}\eta_{G}{\left(a\right)}^{2}$
• Second Zagreb: $M_{2}(G)=\sum_{ab\in E(G)}\eta_{G}(a)\eta_{G}(b)$
• Reduced second Zagreb: $RM_{2}(G)=\sum_{ab\in E(G)}(\eta_{G}(a)-1)(\eta_{G}(b)-1)$
• Hyper Zagerb: $HM(G)=\sum_{ab\in E(G)}{\left[\eta_{G}(a)+\eta_{G}(b)\right]}^{2}$
• Augmented Zagerb: $AZ(G)=\sum_{ab\in E(G)}{\left(\frac{\eta_{G}(a)\eta_{G}(b)}{\eta_{G}(a)+\eta_{G}(b)-2}\right)}^{3}$
• Atom bond connectivity: $ABC(G)=\sum_{ab\in E(G)}\sqrt{\frac{\eta_{G}(a)+\eta_{G}(b)-2}{\eta_{G}(a)\eta_{G}(b)}}$
• Harmonic: $H(G)=\sum_{ab\in E(G)}\frac{2}{\eta_{G}(a)+\eta_{G}(b)}$
• Sum-connectivity: $SC(G)=\sum_{ab\in E(G)}\frac{1}{\sqrt{\eta_{G}(a)+\eta_{G}(b)}}$
• Geometric arithmetic: $GA(G)=\sum_{ab\in E(G)}2\left(\frac{\sqrt{\eta_{G}(a)\eta_{G}(b)}}{\eta_{G}(a)+\eta_{G}(b)}\right)$
• Inverse sum indeg: $ISI(G)=\sum_{ab\in E(G)}\left(\frac{\eta_{G}(a)\eta_{G}(b)}{\eta_{G}(a)+\eta_{G}(b)}\right)$

## 3 Cove-edged graphene nanoribbon

In this section, we explain the exact mathematical formulations for a number of significant distance-based topological indices of CGNR(m,n) for both m≥3n and m<3n with m≡0(mod3), m≡1(mod3), and m≡2(mod3). The molecular structure of CGNR(m,n) comprises of 12mn+6m+6n vertices and 18mn+7m+5n−1 edges. In this section, we derive the expression for CGNR(m,n) by means of the following two theorems.

Theorem 1 *Let* G *be a cove edged graphene nanoribbon of dimension* m,n *. Then, the topological descriptors for* m≥3n *are,*

(i) $W(G)=3(160m^{3}n^{2}+160m^{3}n+40m^{3}+240m^{2}n^{2}+120m^{2}n+360mn^{4}+720mn^{3}+650mn^{2}+170mn+5m-216n^{5}-360n^{4}-340n^{3}-195n^{2}-104n)/5$ *.*
(ii) $W_{e}(G)=(6480m^{3}n^{2}+5040m^{3}n+980m^{3}+540m^{2}n^{2}-2760m^{2}n-1155m^{2}+14580mn^{4}+22680mn^{3}+13980mn^{2}+3630mn+565m-8748n^{5}-12960n^{4}-12810n^{3}-8220n^{2}-2472n-30)/30$ *.*
(iii) $W_{ve}(G)=(1440m^{3}n^{2}+1280m^{3}n+280m^{3}+1140m^{2}n^{2}+120m^{2}n-165m^{2}+3240mn^{4}+5760mn^{3}+4170mn^{2}+940mn+65m-1944n^{5}-3060n^{4}-2940n^{3}-1830n^{2}-726n)/10$ *.*
(iv) $Sz_{b}(G)=3(720m^{3}n^{3}+1000m^{3}n^{2}+460m^{3}n+70m^{3}+1080m^{2}n^{3}+960m^{2}n^{2}+210m^{2}n+120mn^{4}+930mn^{3}+995mn^{2}+335mn+20m-216n^{5}-1200n^{4}-1285n^{3}-255n^{2}-44n)/5$ *.*
(v) $Sz_{e}(G)=(29160m^{3}n^{3}+34020m^{3}n^{2}+13230m^{3}n+1715m^{3}-13230m^{2}n^{2}-10545m^{2}n-2100m^{2}+27540mn^{4}+48510mn^{3}+33840mn^{2}+10245mn+1135m-8748n^{5}-39600n^{4}-41470n^{3}-12090n^{2}-2222n-30)/30$ *.*
(vi) $Sz_{ev}(G)=(3240m^{3}n^{3}+4140m^{3}n^{2}+1750m^{3}n+245m^{3}+2430m^{2}n^{3}+1155m^{2}n^{2}-300m^{2}n-150m^{2}+1800mn^{4}+4305mn^{3}+3585mn^{2}+1085mn+85m-972n^{5}-4380n^{4}-4585n^{3}-1080n^{2}-203n)/5$ *.*
(vii) $PI(G)=324m^{2}n^{2}+252m^{2}n+49m^{2}+90mn^{2}-32mn-27m+72n^{3}+82n^{2}-8n+2$ *.*
(viii) $S(G)=2(1440m^{3}n^{2}+1280m^{3}n+280m^{3}+1680m^{2}n^{2}+600m^{2}n-60m^{2}+3240mn^{4}+5760mn^{3}+4590mn^{2}+1090mn+50m-1944n^{5}-3060n^{4}-2940n^{3}-1755n^{2}-741n)/5$ *.*
(ix) $Gut(G)=(12960m^{3}n^{2}+10080m^{3}n+1960m^{3}+10800m^{2}n^{2}+2040m^{2}n-840m^{2}+29160mn^{4}+45360mn^{3}+32010mn^{2}+7290mn+515m-17496n^{5}-25920n^{4}-24540n^{3}-14835n^{2}-5214n-15)/15$ *.*

**Figure 3:**
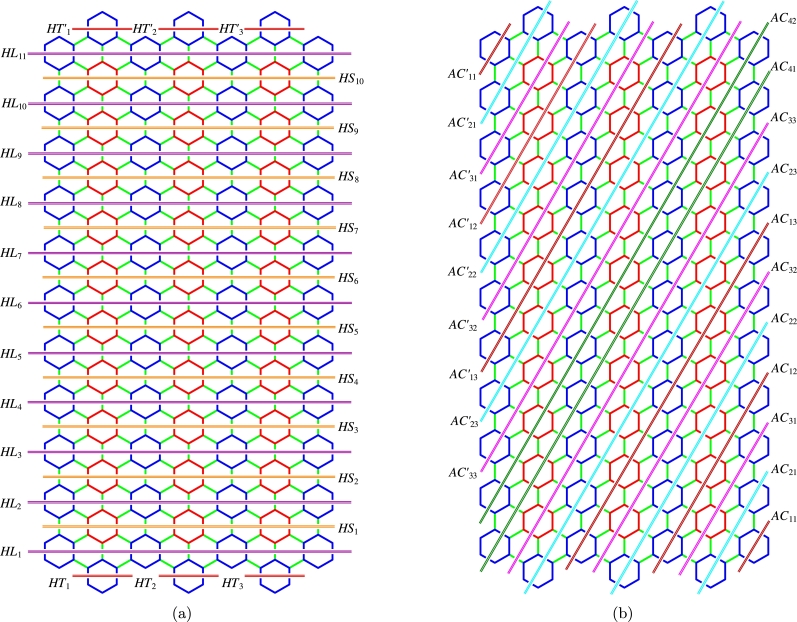
(a) Horizontal cuts; (b) Acute cuts.

**Figure 4:**
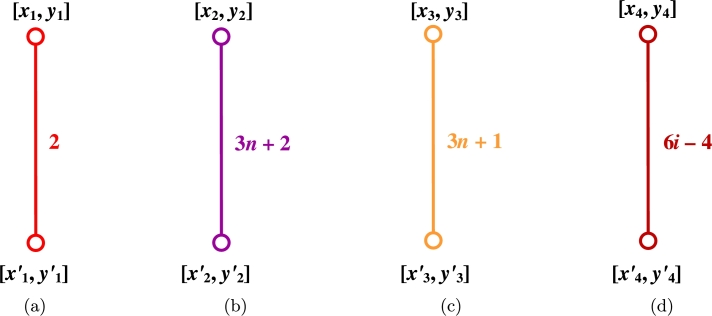
(a) *HT*; (b) $\left\{HL_{i}:i\in N_{m}\right\}$; (c) $\left\{HS_{i}:i\in N_{m-1}\right\}$; (d) $\left\{AC_{1i}:i\in N_{n}\right\}$.

**Figure 5:**
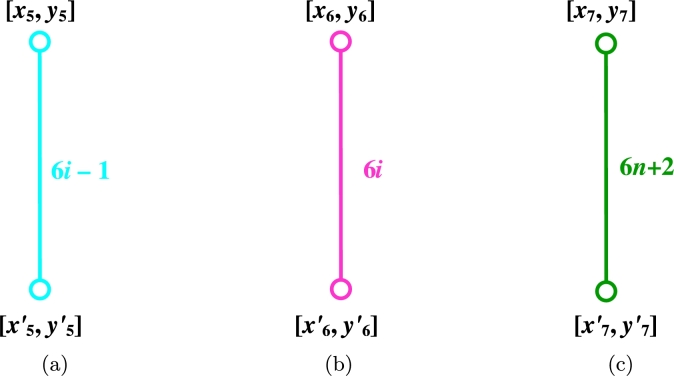
(a) $\left\{AC_{2i}:i\in N_{n}\right\}$; (b) $\left\{AC_{3i}:i\in N_{n}\right\}$; (c) $\left\{AC_{4i}:i\in N_{m-3n}\right\}$.

Proof Let $\left\{HT_{i}:i\in N_{n}\right\}$, $\left\{HT_{i}^{\prime }:i\in N_{n}\right\}$, $\left\{HL_{i}:i\in N_{m}\right\}$, $\left\{HS_{i}:i\in N_{m-1}\right\}$ be the Horizontal Terminal cut, Horizontal Long and Horizontal Short cuts. Also, let $\left\{AC_{1i}:i\in N_{n}\right\}$, $\left\{AC_{1i}^{\prime }:i\in N_{n}\right\}$, $\left\{AC_{2i}:i\in N_{n}\right\}$, $\left\{AC_{2i}^{\prime }:i\in N_{n}\right\}$, $\left\{AC_{3i}:i\in N_{n}\right\}$, $\left\{AC_{3i}^{\prime }:i\in N_{n}\right\}$, and $\left\{AC_{4i}:i\in N_{m-3n}\right\}$ be the various acute cuts of the CGNR(m,n) as shown in Fig. 3(a-b). The quotient graphs $HT_{i}$, $HL_{i}$, $HS_{i}$, $AC_{1i}$, $AC_{2i}$, $AC_{3i}$, and $AC_{4i}$ are $K_{2}$ graph as sketched in Fig. 4(a-d) and 5(a-c), where $\left(x_{i},y_{i}\right)$ and $\left(x_{i}^{\prime },y_{i}^{\prime }\right)$ are number of vertices and edges in each graph components respectively. These values are given in Table 1.

(10) $$W(G)=2nx_{1}x_{1}^{\prime }+\sum_{i=1}^{m}x_{2}x_{2}^{\prime }+\sum_{i=1}^{m-1}x_{3}x_{3}^{\prime }+2[2\sum_{i=1}^{n}x_{4}x_{4}^{\prime }+2\sum_{i=1}^{n}x_{5}x_{5}^{\prime }+2\sum_{i=1}^{n}x_{6}x_{6}^{\prime }+\sum_{i=1}^{m-3n}x_{7}x_{7}^{\prime }].\;$$

(11) $$W_{e}(G)=2ny_{1}y_{1}^{\prime }+\sum_{i=1}^{m}y_{2}y_{2}^{\prime }+\sum_{i=1}^{m-1}y_{3}y_{3}^{\prime }+2[2\sum_{i=1}^{n}y_{4}y_{4}^{\prime }+2\sum_{i=1}^{n}y_{5}y_{5}^{\prime }+2\sum_{i=1}^{n}y_{6}y_{6}^{\prime }+\sum_{i=1}^{m-3n}y_{7}y_{7}^{\prime }].\;$$

(12) $$W_{ve}(G)=\frac{1}{2}[2n(x_{1}y_{1}^{\prime }+x_{1}^{\prime }y_{1})+\sum_{i=1}^{m}(x_{2}y_{2}^{\prime }+x_{2}^{\prime }y_{2})+\sum_{i=1}^{m-1}(x_{3}y_{3}^{\prime }+x_{3}^{\prime }y_{3})+2[2\sum_{i=1}^{n}(x_{4}y_{4}^{\prime }+x_{4}^{\prime }y_{4})\;\;\;\;\;+2\sum_{i=1}^{n}(x_{5}y_{5}^{\prime }+x_{5}^{\prime }y_{5})+2\sum_{i=1}^{n}(x_{6}y_{6}^{\prime }+x_{6}^{\prime }y_{6})+\sum_{i=1}^{m-3n}(x_{7}y_{7}^{\prime }+x_{7}^{\prime }y_{7})]].$$

(13) $$Sz_{v}(G)=4nx_{1}x_{1}^{\prime }+\sum_{i=1}^{m}(3n+2)x_{2}x_{2}^{\prime }+\sum_{i=1}^{m-1}(3n+1)x_{3}x_{3}^{\prime }+2[2\sum_{i=1}^{n}(6i-4)x_{4}x_{4}^{\prime }+2\sum_{i=1}^{n}(6i-1)x_{5}x_{5}^{\prime }\;\;\;\;\;+2\sum_{i=1}^{n}6ix_{6}x_{6}^{\prime }+\sum_{i=1}^{m-3n}(6n+2)x_{7}x_{7}^{\prime }].$$

(14) $$Sz_{e}(G)=4ny_{1}y_{1}^{\prime }+\sum_{i=1}^{m}(3n+2)y_{2}y_{2}^{\prime }+\sum_{i=1}^{m-1}(3n+1)y_{3}y_{3}^{\prime }+2[2\sum_{i=1}^{n}(6i-4)y_{4}y_{4}^{\prime }+2\sum_{i=1}^{n}(6i-1)y_{5}y_{5}^{\prime }\;\;\;\;\;+2\sum_{i=1}^{n}6iy_{6}y_{6}^{\prime }+\sum_{i=1}^{m-3n}(6n+2)y_{7}y_{7}^{\prime }].$$

(15) $$Sz_{ev}(G)=\frac{1}{2}[4n(x_{1}y_{1}^{\prime }+x_{1}^{\prime }y_{1})+\sum_{i=1}^{m}(3n+2)(x_{2}y_{2}^{\prime }+x_{2}^{\prime }y_{2})+\sum_{i=1}^{m-1}(3n+1)(x_{3}y_{3}^{\prime }+x_{3}^{\prime }y_{3})\;\;\;\;\;+2[2\sum_{i=1}^{n}(6i-4)(x_{4}y_{4}^{\prime }+x_{4}^{\prime }y_{4})+2\sum_{i=1}^{n}(6i-1)(x_{5}y_{5}^{\prime }+x_{5}^{\prime }y_{5})+2\sum_{i=1}^{n}6i(x_{6}y_{6}^{\prime }+x_{6}^{\prime }y_{6})\;\;\;\;\;+\sum_{i=1}^{m-3n}(6n+2)(x_{7}y_{7}^{\prime }+x_{7}^{\prime }y_{7})]].$$

(16) $$PI(G)=4n(y_{1}+y_{1}^{\prime })+\sum_{i=1}^{m}(3n+2)(y_{2}+y_{2}^{\prime })+\sum_{i=1}^{m-1}(3n+1)(y_{3}+y_{3}^{\prime })+2[2\sum_{i=1}^{n}(6i-4)(y_{4}+y_{4}^{\prime })\;\;\;\;\;+2\sum_{i=1}^{n}(6i-1)(y_{5}+y_{5}^{\prime })+2\sum_{i=1}^{n}6i(y_{6}+y_{6}^{\prime })+\sum_{i=1}^{m-3n}(6n+2)(y_{7}+y_{7}^{\prime })].$$

(17) $$S(G)=2n[x_{1}^{\prime }(2y_{1}+2)+x_{1}(2y_{1}^{\prime }+2)]+\sum_{i=1}^{m}[x_{2}^{\prime }(2y_{2}+3n+2)+x_{2}(2y_{2}^{\prime }+3n+2)]\;\;\;\;\;+\sum_{i=1}^{m-1}[x_{3}^{\prime }(2y_{3}+3n+1)+x_{3}(2y_{3}^{\prime }+3n+1)]+2[2\sum_{i=1}^{n}[x_{4}^{\prime }(2y_{4}+6i-4)+x_{4}(2y_{4}^{\prime }+6i-4)]\;\;\;\;\;+2\sum_{i=1}^{n}[x_{5}^{\prime }(2y_{5}+6i-1)+x_{5}(2y_{5}^{\prime }+6i-1)]+2\sum_{i=1}^{n}[x_{6}^{\prime }(2y_{6}+6i)+x_{6}(2y_{6}^{\prime }+6i)]\;\;\;\;\;+\sum_{i=1}^{m-3n}[x_{7}^{\prime }(2y_{7}+6n+2)+x_{7}(2y_{7}^{\prime }+6n+2)]].$$

(18) $$Gut(G)=2n(2y_{1}+2)(2y_{1}^{\prime }+2)+\sum_{i=1}^{m}(2y_{2}+3n+2)(2y_{2}^{\prime }+3n+2)+\sum_{i=1}^{m-1}(2y_{3}+3n+1)(2y_{3}^{\prime }+3n+1)\;\;\;\;\;+2[2\sum_{i=1}^{n}(2y_{4}+6i-4)(2y_{4}^{\prime }+6i-4)+2\sum_{i=1}^{n}(2y_{5}+6i-1)(2y_{5}^{\prime }+6i-1)\;\;\;\;\;+2\sum_{i=1}^{n}(2y_{6}+6i)(2y_{6}^{\prime }+6i)+\sum_{i=1}^{m-3n}(2y_{7}+6n+2)(2y_{7}^{\prime }+6n+2)].$$

 □

**Table 1:** *m* ≥ 3*n*.

Quotient Graph	$w_{b}$	$s_{b}$
**Table:** G/HT	**Table:** *x*_1_ = 3 $x_{1}^{\prime }=|V(G)|-x_{1}$	**Table:** *y*_1_ = 2 $y_{1}^{\prime }=|E(G)|-y_{1}-2$
**Table:** $G/HL_{i}$ $i\in N_{m}$	**Table:** *x*_2_ = 12*ni* + 6*i* − 3*n* − 3 $x_{2}^{\prime }=|V(G)|-x_{2}$	**Table:** *y*_2_ = 18*ni* + 7*i* − 8*n* − 5 $y_{2}^{\prime }=|E(G)|-y_{2}-(3n+2)$
**Table:** $G/HS_{i}$ $i\in N_{m-1}$	**Table:** *x*_3_ = 12*ni* + 6*i* + 3*n* $x_{3}^{\prime }=|V(G)|-x_{3}$	**Table:** *y*_3_ = 18*ni* + 7*i* + *n* − 1 $y_{3}^{\prime }=|E(G)|-y_{3}-(3n+1)$
**Table:** $G/AC_{1i}$ $i\in N_{n}$	**Table:** *x*_4_ = 18*i*^2^ − 18*i* + 3 $x_{4}^{\prime }=|V(G)|-x_{4}$	**Table:** *y*_4_ = 27*i*^2^ − 35*i* + 10 $y_{4}^{\prime }=|E(G)|-y_{4}-(6i-4)$
**Table:** $G/AC_{2i}$ $i\in N_{n}$	**Table:** *x*_5_ = 18*i*^2^ − 6*i* $x_{5}^{\prime }=|V(G)|-x_{5}$	**Table:** *y*_5_ = 27*i*^2^ − 17*i* + 2 $y_{5}^{\prime }=|E(G)|-y_{5}-(6i-1)$
**Table:** $G/AC_{3i}$ $i\in N_{n}$	**Table:** *x*_6_ = 18*i*^2^ + 6*i* $x_{6}^{\prime }=|V(G)|-x_{6}$	**Table:** *y*_6_ = 27*i*^2^ + *i* $y_{6}^{\prime }=|E(G)|-y_{6}-6i$
**Table:** $G/AC_{4i}$ $i\in N_{m-3n}$	**Table:** *x*_7_ = 18*n*^2^ + 6*n* + 12*ni* + 6*i* − 3 $x_{7}^{\prime }=|V(G)|-x_{7}$	**Table:** *y*_7_ = 27*n*^2^ + *n* + 18*ni* + 7*i* − 5 $y_{7}^{\prime }=|E(G)|-y_{7}-(6n+2)$

Equations (10)-(18) are derived from Equations (1)-(9). Following the computational procedure adopted in Theorem 1, we present the values of various distance-based topological indices of CGNR(m,n) for the case m<3n.

Theorem 2 *Let*G*be a*CGNR*,*m<3n*. Then*$TI(G)=TI(G_{1})+\left\{ \begin{array}{ccc} TI(G_{A})\; & :\; & m\equiv 0(\mathrm{mod}\;3)\\ TI(G_{B})\; & :\; & m\equiv 1(\mathrm{mod}\;3)\\ TI(G_{C})\; & :\; & m\equiv 2(\mathrm{mod}\;3) \end{array}\right.$

*where we use*

(i) *Wiener*
  • $W(G_{1})=(-24m^{5}+360m^{4}n+120m^{4}+2160m^{3}n^{2}+2880m^{3}n+680m^{3}+6480m^{2}n^{3}+12960m^{2}n^{2}+6840m^{2}n+720m^{2}+6480mn^{3}+6750mn^{2}+1620n^{3}+270mn-135m+1215n^{2}-810n)/45$*.*
  • $W(G_{A})=(-126m-810n)/45$*.*
  • $W(G_{B})=(-186m-630n+40)/45$*.*
  • $W(G_{C})=(-126m-810n-40)/45$*.*
(ii) *Edge-Wiener*
  • $W_{e}(G_{1})=(-324m^{5}+4860m^{4}n+1440m^{4}+29160m^{3}n^{2}+28080m^{3}n+4300m^{3}+87480m^{2}n^{3}+82620m^{2}n^{2}+15840m^{2}n-2355m^{2}+48600mn^{3}+3780mn^{2}-15570mn+1665m+6750n^{3}-1620n^{2}-4860n-270)/270$*.*
  • $W_{e}(G_{A})=(-756m-4860n)/270$*.*
  • $W_{e}(G_{B})=(-1296m-3240n+80)/270$*.*
  • $W_{e}(G_{C})=(-816m-4680n-200)/270$*.*
(iii) *Vertex-Edge-Wiener*
  • $W_{ve}(G_{1})=(-72m^{5}+1080m^{4}n+340m^{4}+6480m^{3}n^{2}+7440m^{3}n+1440m^{3}+19440m^{2}n^{3}+28620m^{2}n^{2}+10800m^{2}n+435m^{2}+15120mn^{3}+8370mn^{2}-3060mn-45m+2700n^{3}+810n^{2}-1620n)/90$*.*
  • $W_{ve}(G_{A})=(-378m-1890n)/90$
  • $W_{ve}(G_{B})=(-538m-1410n+60)/90$*.*
  • $W_{ve}(G_{C})=(-398m-1830n-120)/90$*.*
(iv) *Vertex-Szeged*
  • $Sz_{b}(G_{1})=(56m^{5}-600m^{4}n-400m^{4}+19440m^{3}n^{3}+29160m^{3}n^{2}+14820m^{3}n+2050m^{3}+27000m^{2}n^{3}+25920m^{2}n^{2}+8550m^{2}n+3510mn^{3}+5265mn^{2}+2025mn-135n^{3}+2835n^{2}-1620n)/45$*.*
  • $Sz_{v}(G_{A})=(720m^{2}+1620mn+504m+540n)/45$*.*
  • $Sz_{v}(G_{B})=(1000m^{2}+780mn+64m-60n-340)/45$*.*
  • $Sz_{v}(G_{C})=(560m^{2}+2100mn+884m+600n+400)/45$*.*
(v) *Edge-Szeged*
  • $Sz_{e}(G_{1})=(1188m^{5}+8100m^{4}n-5280m^{4}+787320m^{3}n^{3}+772740m^{3}n^{2}+326250m^{3}n+36105m^{3}+554040m^{2}n^{3}+89910m^{2}n^{2}-83835m^{2}n-22680m^{2}+33210mn^{3}+68040mn^{2}+62775mn+10125m+11070n^{3}+36450n^{2}-33210n-810)/810$*.*
  • $Sz_{e}(G_{A})=(15120m^{2}n+360m^{2}-87480mn^{2}-2160mn+6912m-24300n^{2}+14040n)/810$*.*
  • $Sz_{e}(G_{B})=(5400m^{2}n+7380m^{2}-58320mn^{2}-25920mn-6348m-16200n^{2}-900n-240)/810$*.*
  • $Sz_{e}(G_{C})=(5400m^{2}n-4860m^{2}-58320mn^{2}-28080mn+18012m-16200n^{2}-3420n+16440)/810$*.*
(vi) *Edge-Vertex-Szeged*
  • $Sz_{ev}(G_{1})=(192m^{5}-900m^{4}n-980m^{4}+87480m^{3}n^{3}+108540m^{3}n^{2}+49530m^{3}n+6175m^{3}+91530m^{2}n^{3}+57105m^{2}n^{2}-810m^{2}n-1620m^{2}+4995mn^{3}+5265mn^{2}+6075mn+405m+1485n^{3}+8100n^{2}-5265n)/135$*.*
  • $Sz_{ev}(G_{A})=(10890m^{2}n+1080m^{2}-810mn^{2}+1080mn+1368m-2430n^{2}+1350n)/135$*.*
  • $Sz_{ev}(G_{B})=(10350m^{2}n+1950m^{2}+810mn^{2}-1800mn-502m-1620n^{2}-480n-760)/135$*.*
  • $Sz_{ev}(G_{C})=(10350m^{2}n+270m^{2}+810mn^{2}+1080mn+2218m-1620n^{2}+120n+1600)/135$*.*
(vii) *Padmakar-Ivan*
  • $PI(G_{1})=(8m^{3}+972m^{2}n^{2}+684m^{2}n+131m^{2}+486mn^{2}-45m+102n^{2}-24n+6)/3$*.*
  • $PI(G_{A})=(-24m-36n)/3$*.*
  • $PI(G_{B})=(-28m-24n)/3$*.*
  • $PI(G_{C})=(-28m-24n-16)/3$*.*
(viii) *Schultz*
  • $S(G_{1})=(-432m^{5}+6480m^{4}n+2040m^{4}+38880m^{3}n^{2}+44640m^{3}n+8640m^{3}+116640m^{2}n^{3}+200880m^{2}n^{2}+90720m^{2}n+8280m^{2}+90720mn^{3}+72900mn^{2}-10260mn-1080m+16200n^{3}+8910n^{2}-10530n)/135$*.*
  • $S(G_{A})=(-2268m-11340n)/135$*.*
  • $S(G_{B})=(-3228m-8460n+360)/135$*.*
  • $S(G_{C})=(-2388m-10980n-720)/135$*.*
(ix) *Gutman*
  • $Gut(G_{1})=(-648m^{5}+9720m^{4}n+2880m^{4}+58320m^{3}n^{2}+56160m^{3}n+8960m^{3}+174960m^{2}n^{3}+252720m^{2}n^{2}+96480m^{2}n+7800m^{2}+97200mn^{3}+53730mn^{2}-26550mn-45m+13500n^{3}+4725n^{2}-11610n-135)/135$*.*
  • $Gut(G_{A})=(-3132m-11880n)/135$*.*
  • $Gut(G_{B})=(-4392m-8100n+160)/135$*.*
  • $Gut(G_{C})=(-3432m-10980n-1120)/135$*.*

Proof Let $\left\{HT_{i}:i\in N_{n}\right\}$, $\left\{HT_{i}^{\prime }:i\in N_{n}\right\}$, $\left\{HL_{i}:i\in N_{m}\right\}$, $\left\{HS_{i}:i\in N_{m-1}\right\}$ be the various horizontal cuts as discussed in Theorem 1. When m≡0(mod3), let $\left\{AC_{1i}:i\in N_{\frac{m}{3}}\right\}$, $\left\{AC_{2i}:i\in N_{\frac{m}{3}}\right\}$ and $\left\{AC_{3i}:i\in N_{\frac{m}{3}}\right\}$ be the first set of three acute cuts symmetrical with $\left\{AC_{1i}^{\prime }:i\in N_{\frac{m}{3}}\right\}$, $\left\{AC_{2i}^{\prime }:i\in N_{\frac{m}{3}}\right\}$ and $\left\{AC_{3i}^{\prime }:i\in N_{\frac{m}{3}}\right\}$. In addition, let $\left\{AC_{4i}:i\in N_{\frac{3n-m}{3}}\right\}$, $\left\{AC_{5i}:i\in N_{\frac{3n-m}{3}}\right\}$ and $\left\{AC_{6i}:i\in N_{\frac{3n-m}{3}}\right\}$ be the second set of three acute cuts. In the case of obtuse cuts, we have the same number of cuts as in acute cuts.

**Figure 6:**
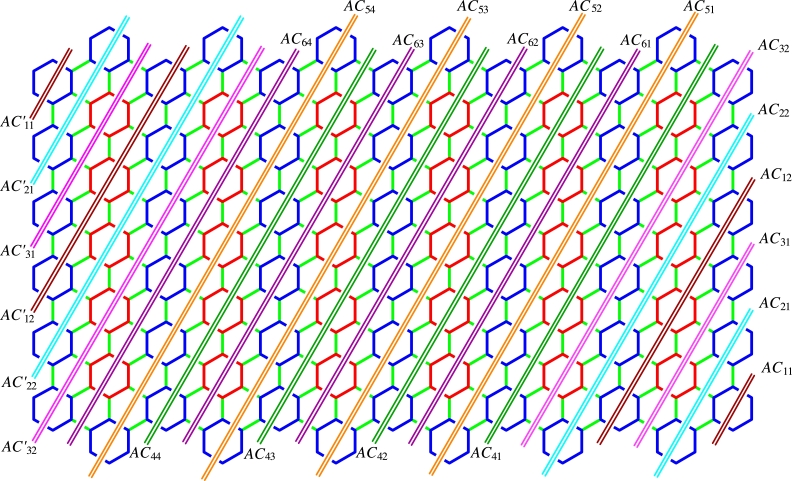
Various acute cuts of *CGNR*(*m*,*n*), *m* < 3*n*.

For other congruence classes see Fig. 6 and for their values, refer Table 2, Table 3, Table 4. □

**Table 2:** *m* < 3*n*, *m* ≡ 0(mod 3).

Quotient Graph	*w*_*b*_	*s*_*b*_
**Table:** $G/AC_{1i}$ $i\in N_{\frac{m}{3}}$	**Table:** *x*_4_ = 18*i*^2^ − 18*i* + 3 $x_{4}^{\prime }=|V(G)|-x_{4}$	**Table:** *y*_4_ = 27*i*^2^ − 35*i* + 10 $y_{4}^{\prime }=|E(G)|-y_{4}-(6i-4)$
**Table:** $G/AC_{2i}$ $i\in N_{\frac{m}{3}}$	**Table:** *x*_5_ = 18*i*^2^ − 6*i* $x_{5}^{\prime }=|V(G)|-x_{5}$	**Table:** *y*_5_ = 27*i*^2^ − 17*i* + 2 $y_{5}^{\prime }=|E(G)|-y_{5}-(6i-1)$
**Table:** $G/AC_{3i}$ $i\in N_{\frac{m}{3}}$	**Table:** *x*_6_ = 18*i*^2^ + 6*i* $x_{6}^{\prime }=|V(G)|-x_{6}$	**Table:** *y*_6_ = 27*i*^2^ + *i* $y_{6}^{\prime }=|E(G)|-y_{6}-6i$
**Table:** $G/AC_{4i}$ $i\in N_{\frac{3n-m}{3}}$	**Table:** *x*_7_ = 2*m*^2^ + 12*mi* − 6*m* + 6*i* − 6 $x_{7}^{\prime }=|V(G)|-x_{7}$	**Table:** $y_{7}=\frac{1}{3}[9m^{2}+54mi-35m+15i-18]$ $y_{7}^{\prime }=|E(G)|-y_{7}-2m$
**Table:** $G/AC_{5i}$ $i\in N_{\frac{3n-m}{3}}$	**Table:** *x*_8_ = 2*m*^2^ + 12*mi* − 2*m* + 6*i* − 3 $x_{8}^{\prime }=|V(G)|-x_{8}$	**Table:** $y_{8}=\frac{1}{3}[9m^{2}+54mi-17m+15i-12]$ $y_{8}^{\prime }=|E(G)|-y_{8}-(2m+2)$
**Table:** $G/AC_{6i}$ $i\in N_{\frac{3n-m}{3}}$	**Table:** *x*_9_ = 2*m*^2^ + 12*mi* + 2*m* + 6*i* $x_{9}^{\prime }=|V(G)|-x_{9}$	**Table:** $y_{9}=\frac{1}{3}[9m^{2}+54mi+m+15i]$ $y_{9}^{\prime }=|E(G)|-y_{9}-2m$

**Table 3:** *m* < 3*n*, *m* ≡ 1(mod 3).

Quotient Graph	*w*_*b*_	*s*_*b*_
**Table:** $G/AC_{1i}$ $i\in N_{\frac{m+2}{3}}$	**Table:** *x*_4_ = 18*i*^2^ − 18*i* + 3 $x_{4}^{\prime }=|V(G)|-x_{4}$	**Table:** *y*_4_ = 27*i*^2^ − 35*i* + 10 $y_{4}^{\prime }=|E(G)|-y_{4}-(6i-4)$
**Table:** $G/AC_{2i}$ $i\in N_{\frac{m-1}{3}}$	**Table:** *x*_5_ = 18*i*^2^ − 6*i* $x_{5}^{\prime }=|V(G)|-x_{5}$	**Table:** *y*_5_ = 27*i*^2^ − 17*i* + 2 $y_{5}^{\prime }=|E(G)|-y_{5}-(6i-1)$
**Table:** $G/AC_{3i}$ $i\in N_{\frac{m-1}{3}}$	**Table:** *x*_6_ = 18*i*^2^ + 6*i* $x_{6}^{\prime }=|V(G)|-x_{6}$	**Table:** *y*_6_ = 27*i*^2^ + *i* $y_{6}^{\prime }=|E(G)|-y_{6}-6i$
**Table:** $G/AC_{4i}$ $i\in N_{\frac{3n-m+1}{3}}$	**Table:** *x*_7_ = 2*m*^2^ + 12*mi* − 6*m* + 6*i* − 5 $x_{7}^{\prime }=|V(G)|-x_{7}$	**Table:** $y_{7}=\frac{1}{3}\left[9m^{2}+54mi-35m+15i-16\right]$ $y_{7}^{\prime }=|E(G)|-y_{7}-(2m+1)$
**Table:** $G/AC_{5i}$ $i\in N_{\frac{3n-m+1}{3}}$	**Table:** *x*_8_ = 2*m*^2^ + 12*mi* − 2*m* + 6*i* − 3 $x_{8}^{\prime }=|V(G)|-x_{8}$	**Table:** $y_{8}=\frac{1}{3}\left[9m^{2}+54mi-17m+15i-10\right]$ $y_{8}^{\prime }=|E(G)|-y_{8}-(2m+1)$
**Table:** $G/AC_{6i}$ $i\in N_{\frac{3n-m-2}{3}}$	**Table:** *x*_9_ = 2*m*^2^ + 12*mi* + 2*m* + 6*i* − 1 $x_{9}^{\prime }=|V(G)|-x_{9}$	**Table:** $y_{9}=\frac{1}{3}\left[9m^{2}+54mi+m+15i-4\right]$ $y_{9}^{\prime }=|E(G)|-y_{9}-2m$

**Table 4:** *m* < 3*n*, *m* ≡ 2(mod 3).

Quotient Graph	*w*_*b*_	*s*_*b*_
**Table:** $G/AC_{1i}$ $i\in N_{\frac{m+1}{3}}$	**Table:** *x*_4_ = 18*i*^2^ − 18*i* + 3 $x_{4}^{\prime }=|V(G)|-x_{4}$	**Table:** *y*_4_ = 27*i*^2^ − 35*i* + 10 $y_{4}^{\prime }=|E(G)|-y_{4}-(6i-4)$
**Table:** $G/AC_{2i}$ $i\in N_{\frac{m+1}{3}}$	**Table:** *x*_5_ = 18*i*^2^ − 6*i* $x_{5}^{\prime }=|V(G)|-x_{5}$	**Table:** *y*_5_ = 27*i*^2^ − 17*i* + 2 $y_{5}^{\prime }=|E(G)|-y_{5}-(6i-1)$
**Table:** $G/AC_{3i}$ $i\in N_{\frac{m-2}{3}}$	**Table:** *x*_6_ = 18*i*^2^ + 6*i* $x_{6}^{\prime }=|V(G)|-x_{6}$	**Table:** *y*_6_ = 27*i*^2^ + *i* $y_{6}^{\prime }=|E(G)|-y_{6}-6i$
**Table:** $G/AC_{4i}$ $i\in N_{\frac{3n-m+2}{3}}$	**Table:** *x*_7_ = 2*m*^2^ + 12*mi* − 6*m* + 6*i* − 5 $x_{7}^{\prime }=|V(G)|-x_{7}$	**Table:** $y_{7}=\frac{1}{3}\left[9m^{2}+54mi-35m+15i-14\right]$ $y_{7}^{\prime }=|E(G)|-y_{7}-2m$
**Table:** $G/AC_{5i}$ $i\in N_{\frac{3n-m-1}{3}}$	**Table:** *x*_8_ = 2*m*^2^ + 12*mi* − 2*m* + 6*i* − 4 $x_{8}^{\prime }=|V(G)|-x_{8}$	**Table:** $y_{8}=\frac{1}{3}\left[9m^{2}+54mi-17m+15i-14\right]$ $y_{8}^{\prime }=|E(G)|-y_{8}-(2m+1)$
**Table:** $G/AC_{6i}$ $i\in N_{\frac{3n-m-1}{3}}$	**Table:** *x*_9_ = 2*m*^2^ + 12*mi* + 2*m* + 6*i* $x_{9}^{\prime }=|V(G)|-x_{9}$	**Table:** $y_{9}=\frac{1}{3}\left[9m^{2}+54mi+m+15i-2\right]$ $y_{9}^{\prime }=|E(G)|-y_{9}-(2m+1)$

**Figure 7:**
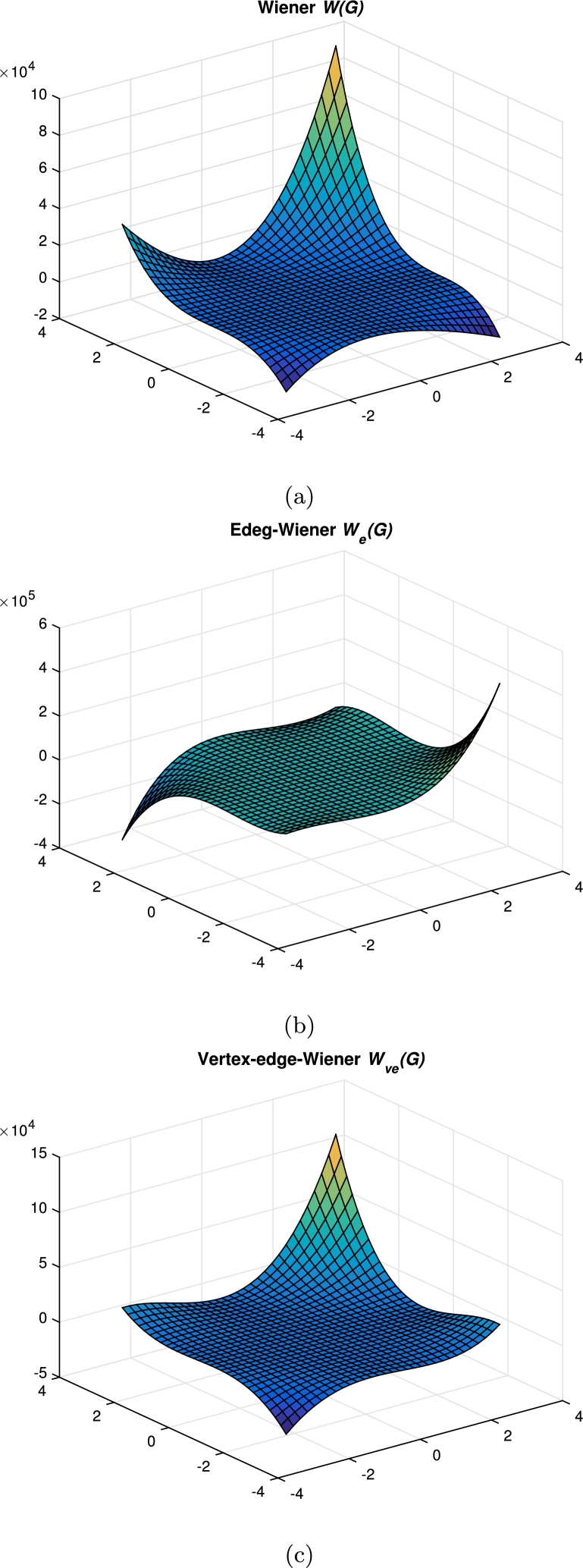
Graphical representation of (a) Wiener; (b) Edge-Wiener; (c) Vertex-edge Wiener.

**Figure 8:**
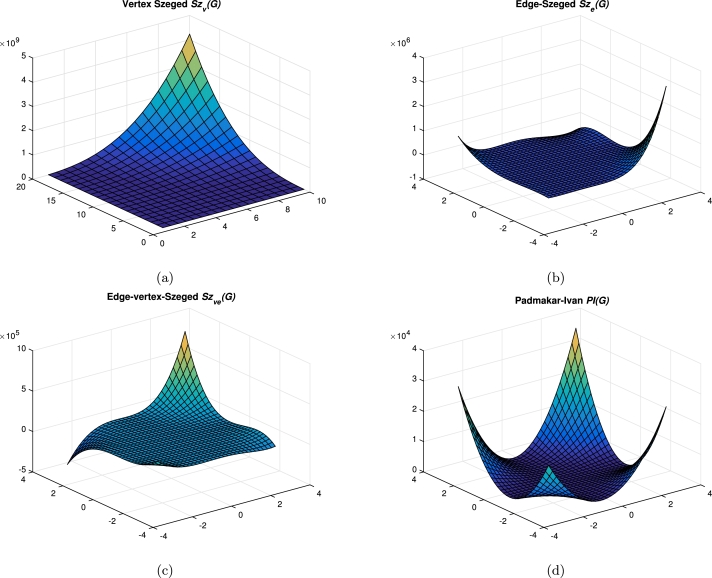
Graphical representation of (a) Vertex Szeged; (b) Edge-Szeged; (c) Edge-vertex-Szeged; (d) Padmakar-Ivan.

**Figure 9:**
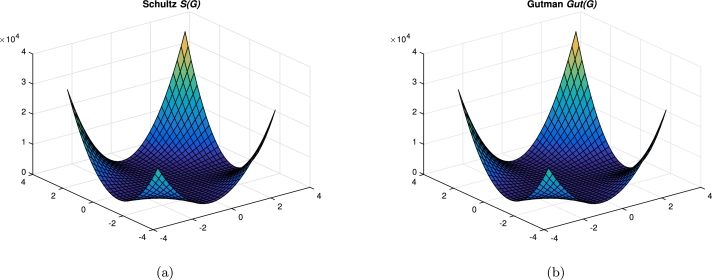
Graphical representation of (a) Schultz; (b) Gutman.

**Figure 10:**
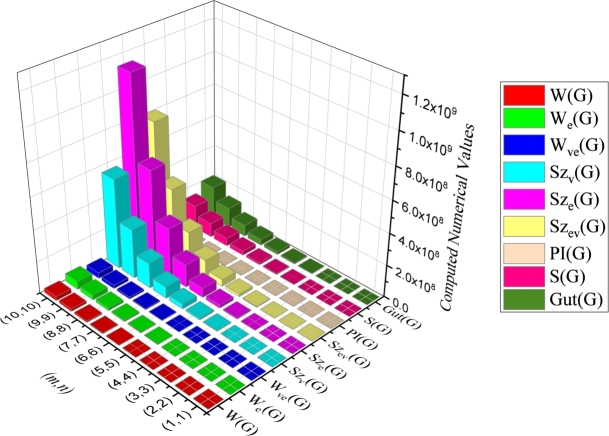
Graphical representation of distance-based topological indices of *CGNR*(*m*,*n*).

This section concludes with a graphical comparison of topological indices of CGNR as shown in Figs. 7(a-c), 8(a-d) and 9(a-b). Also the numerical values presented in Tables 5 & 6 are depicted in Fig. 10.

**Table 5:** Computed numerical value for distance-based indices.

(*m*,*n*)	*W*(*G*)	*W*_*e*_(*G*)	*W*_*ve*_(*G*)	*Sz*_*v*_(*G*)	*Sz*_*e*_(*G*)
(1,1)	1020	1014	1020	3272	2973
(2,2)	17221	24365	20544	86661	122180
(3,3)	98595	159485	125614	706013	1148590
(4,4)	354973	617808	468819	3311095	5798504
(5,5)	979960	1786622	1324197	11271514	20661107
(6,6)	2274876	4282931	3123144	31113656	58845848
(7,7)	4672688	9007302	6490311	74068683	143329654
(8,8)	8761945	17197730	12279514	157931558	311003986
(9,9)	15310720	30483489	21609635	309231119	617421734
(10,10)	25290537	50938988	35900526	565711182	1142243956

**Table 6:** Computed numerical value for distance-based indices.

(*m*,*n*)	$Sz_{ev}(G)$	PI(G)	S(G)	Gut(G)
(1,1)	3353	713	4775	5595
(2,2)	104787	8477	89016	114931
(3,3)	907228	37309	530825	714010
(4,4)	4398567	109003	1955675	2692395
(5,5)	15295277	253133	5480030	7658624
(6,6)	42852586	507047	12854952	18155634
(7,7)	103141207	915867	26609724	37876189
(8,8)	221790627	1532495	50195464	71878298
(9,9)	437198959	2417607	88128739	126800644
(10,10)	804209352	3639653	146135184	211078005

## 4 Degree-based indices

This part of the paper gives the mathematical derivations of the degree-based TIs of CGNR based on the edge-partition of degree of end vertices that reported in Table 7.

**Table 7:** The edge partition of *CGNR*(*m*,*n*).

S. No	Edge Type	$\left(\eta_{G}(a),\eta_{G}(b)\right)$	Frequency
1	$E_{1}$	(2,2)	2*m* + 4*n* + 4
2	$E_{2}$	(2,3)	4*m* + 8*n* − 4
3	$E_{3}$	(3,3)	18*mn* + *m* − 7*n* − 1

Theorem 3 *Let*G*be a*CGNR(m,n)*. Then*

(i) $R(G)=(18mn+(4+2\sqrt{6})m+(4\sqrt{6}-1)n+5-2\sqrt{6})/3$*.*
(ii) $RR(G)=54mn+(7+4\sqrt{6})m+(8\sqrt{6}-13)n+5-4\sqrt{6}$*.*
(iii) $RRR(G)=36mn+(4+4\sqrt{2})m+(8\sqrt{2}-10)n+2-4\sqrt{2}$*.*
(iv) $M_{1}(G)=108mn+34m+14n-10$*.*
(v) $M_{2}(G)=162mn+41m+n-17$*.*
(vi) $RM_{2}(G)=72mn+14m-8n-8$*.*
(vii) HM(G)=648mn+168m+12n−72*.*
(viii) AZ(G)=(13122mn+3801m+1041n−729)/64*.*
(ix) $ABC(G)=(36mn+(9\sqrt{2}+2)m-14n+18\sqrt{2}n-2)/3$*.*
(x) H(G)=(90mn+44m+43n+1)/15*.*
(xi) $SC(G)=(90mn\sqrt{6}+(5\sqrt{6}+24\sqrt{5}+30)m+(6-33\sqrt{6})n+60-6\sqrt{6})/30$*.*
(xii) $GA(G)=(90mn+(15+8\sqrt{6})m+(16\sqrt{6}-15)n-8\sqrt{6}+15)/5$*.*
(xiii) ISI(G)=(270mn+83m+31n−23)/10*.*

**Figure 11:**
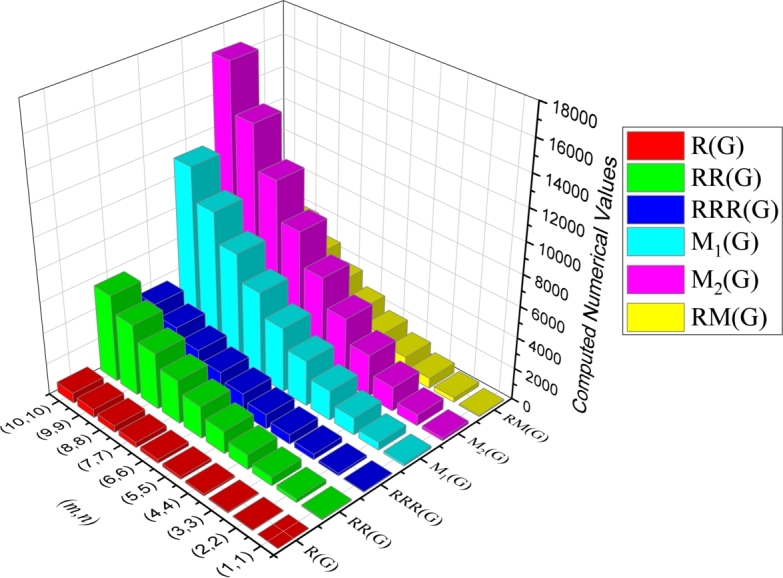
Graphical representation of degree-based topological indices of *CGNR*(*m*,*n*).

**Figure 12:**
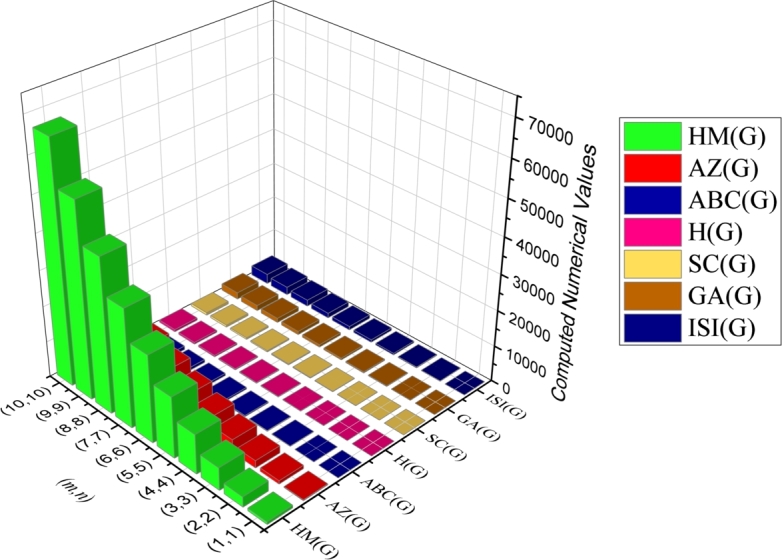
Graphical representation of degree-based topological indices of *CGNR*(*m*,*n*).

The graphical representation of various degree-based topological indices of CGNR(m,n) is depicted in Fig. 11, Fig. 12 and presented in numerical values in Tables 8 & 9.

**Table 8:** Computed numerical value for degree-based indices.

(*m*,*n*)	*R*(*G*)	*RR*(*G*)	*RRR*(*G*)	*M*_1_(*G*)	*M*_1_(*G*)	*RM*(*G*)
(1,1)	12	73	43	146	187	70
(2,2)	36	258	162	518	715	292
(3,3)	72	551	353	1106	1567	658
(4,4)	120	953	616	1910	2743	1168
(5,5)	180	1462	951	2930	4243	1822
(6,6)	251	2080	1358	4166	6067	2620
(7,7)	335	2805	1837	5618	8215	3562
(8,8)	431	3638	2388	7286	10687	4648
(9,9)	539	4580	3011	9170	13483	5878
(10,10)	659	5629	3706	11270	16603	7252

**Table 9:** Computed numerical value for degree-based indices.

(*m*,*n*)	*HM*(*G*)	*AZ*(*G*)	*ABC*(*G*)	*H*(*G*)	*SC*(*G*)	*GA*(*G*)	*ISI*(*G*)
(1,1)	756	269	20	12	10	29	36
(2,2)	2880	960	65	36	32	95	129
(3,3)	6300	2061	134	71	70	196	275
(4,4)	11016	3572	226	119	122	334	475
(5,5)	17028	5493	343	179	189	508	730
(6,6)	24336	7824	484	251	270	718	1038
(7,7)	32940	10565	648	335	366	963	1401
(8,8)	42840	13716	837	430	477	1245	1817
(9,9)	54036	17277	1050	538	603	1563	2287
(10,10)	66528	21248	1287	658	743	1917	2812

## 5 Conclusion and future direction

Graphene nanoribbons hold great promise for advancing nanoscale technologies due to their unique combination of electronic, mechanical, and thermal properties. Continued research and development are expected to unlock new applications and improve the scalability and control of GNR production. The topological indices provide information about the structural characteristics of the compound [64]. Therefore, the results obtained in this study are crucial for comprehending the importance of these large-sized aromatic compounds in various fields such as drug discovery, materials science, predictive toxicology, and astrochemistry. The expressions outlined in this article would aid in reducing the extent of repetitive laboratory tasks necessary for studying the physicochemical characteristics of graphene nanoribbons with curved edges. The future researcher could take up the other types of graphenes which has different type of fencing in their molecular structure.

## CRediT authorship contribution statement

**S. Prabhu:** Validation, Supervision, Methodology, Investigation, Conceptualization. **G. Murugan:** Writing – original draft, Software, Investigation. **Muhammad Imran:** Validation, Supervision, Conceptualization. **Micheal Arockiaraj:** Supervision, Methodology, Formal analysis. **Mohammad Mahtab Alam:** Methodology, Investigation, Conceptualization. **Muhammad Usman Ghani:** Validation, Resources, Methodology.

## Declaration of Competing Interest

The authors declare that they have no known competing financial interests or personal relationships that could have appeared to influence the work reported in this paper.

## Data Availability

There is no data associated with this manuscript.
